# Individual-based modeling reveals that the COVID-19 isolation period can be shortened by community vaccination

**DOI:** 10.1038/s41598-022-21645-y

**Published:** 2022-10-20

**Authors:** Chayanin Sararat, Jidchanok Wangkanai, Chaiwat Wilasang, Tanakorn Chantanasaro, Charin Modchang

**Affiliations:** 1 Biophysics Group, Department of Physics, Faculty of Science, Mahidol University, Bangkok, 10400 Thailand; 2 Center for Disease Modeling, Faculty of Science, Mahidol University, Bangkok, 10400 Thailand; 3 Centre of Excellence in Mathematics, MHESI, Bangkok, 10400 Thailand; 4 Thailand Center of Excellence in Physics, Ministry of Higher Education, Science, Research and Innovation, 328 Si Ayutthaya Road, Bangkok, 10400 Thailand

**Keywords:** Applied mathematics, Computational models, Viral infection

## Abstract

The isolation of infected individuals and quarantine of their contacts are usually employed to mitigate the transmission of SARS-CoV-2. Although 14-day isolation of infected individuals could effectively reduce the risk of subsequent transmission, it also substantially impacts the patient's psychological and emotional well-being. It is, therefore, vital to investigate how the isolation duration could be shortened when effective vaccines are available. Here, an individual-based modeling approach was employed to estimate the likelihood of secondary infections and the likelihood of an outbreak following the isolation of a primary case for a range of isolation periods. Our individual-based model integrated the viral loads and infectiousness profiles of vaccinated and unvaccinated infected individuals. The effects of waning vaccine-induced immunity against infection were also considered. By simulating the transmission of the SARS-CoV-2 Delta (B.1.617.2) variant in a community, we found that in the baseline scenario in which all individuals were unvaccinated and nonpharmaceutical interventions were not used, there was an approximately 3% chance that an unvaccinated individual would lead to at least one secondary infection after being isolated for 14 days, and a sustained chain of transmission could occur with a less than 1% chance. With the outbreak risk equivalent to that of the 14-day isolation in the baseline scenario, we found that the isolation duration could be shortened to 7.33 days (95% CI 6.68–7.98) if 75% of people in the community were fully vaccinated with the BNT162b2 vaccine within the last three months. In the best-case scenario in which all individuals in the community are fully vaccinated, isolation of Delta variant-infected individuals may no longer be necessary. However, to keep the outbreak risk lower than 1%, a booster vaccination may be necessary three months after full vaccination.

## Introduction

SARS-CoV-2 spread rapidly throughout the world, causing over 288.23 million infections and 5.48 million deaths by the end of 2021^1^. During the early phase of transmission when vaccines were unavailable, nonpharmaceutical interventions were frontline measures to mitigate transmission^2–5^. Isolation of infected individuals is a critical strategy that is widely employed to break the transmission chain. Institution-based isolation of confirmed cases has been shown to delay the epidemic's peak and reduce the epidemic's size by approximately 57% in a modeling study^6^. Isolation, however, will be effective only if it can be promptly employed to prevent presymptomatic and asymptomatic transmission^7^. In addition, the isolation period should be long enough to ensure that the infected individuals do not spread the disease after isolation. However, although prolonged isolation may reduce the risk of transmission more effectively, it also substantially impacts the patient's financial, psychological, and emotional well-being^8–10^.

COVID-19 vaccines were first made available at the end of 2020^11^, and they have been shown to be effective at preventing infection and transmission^12–14^. Despite the fact that infections can occur even after full vaccination, faster viral clearance was observed in breakthrough infections, indicating that an individual with a breakthrough infection may have a shorter duration of infectiousness^15,16^. This result suggests that those who have been vaccinated may require a shorter period of isolation. It is vital to comprehend how the isolation period could be reduced based on vaccine effectiveness, particularly when we desire to return to normalcy and live with COVID-19 without quarantine and isolation measures.

In this study, we used an individual-based modeling approach to assess the likelihood of secondary infections and the likelihood of an outbreak following the isolation of a BNT162b2 fully vaccinated, Delta variant-infected primary case for a range of isolation periods. Our individual-based model accounted for transmission heterogeneity, variation in the course of infection, and the disease's infectivity profiles in both vaccinated and unvaccinated infected individuals. The effects of different levels of community vaccination coverage using the BNT162b2 vaccine on the likelihood of post-isolation transmission of the SARS-CoV-2 Delta variant were investigated. In addition, the effects of waning vaccine-induced immunity and delays in isolating infected individuals in the community were also examined.

## Methods

### Estimation of infectiousness profiles for vaccine breakthrough infections and vaccine effectiveness against transmission

**Figure 1:**
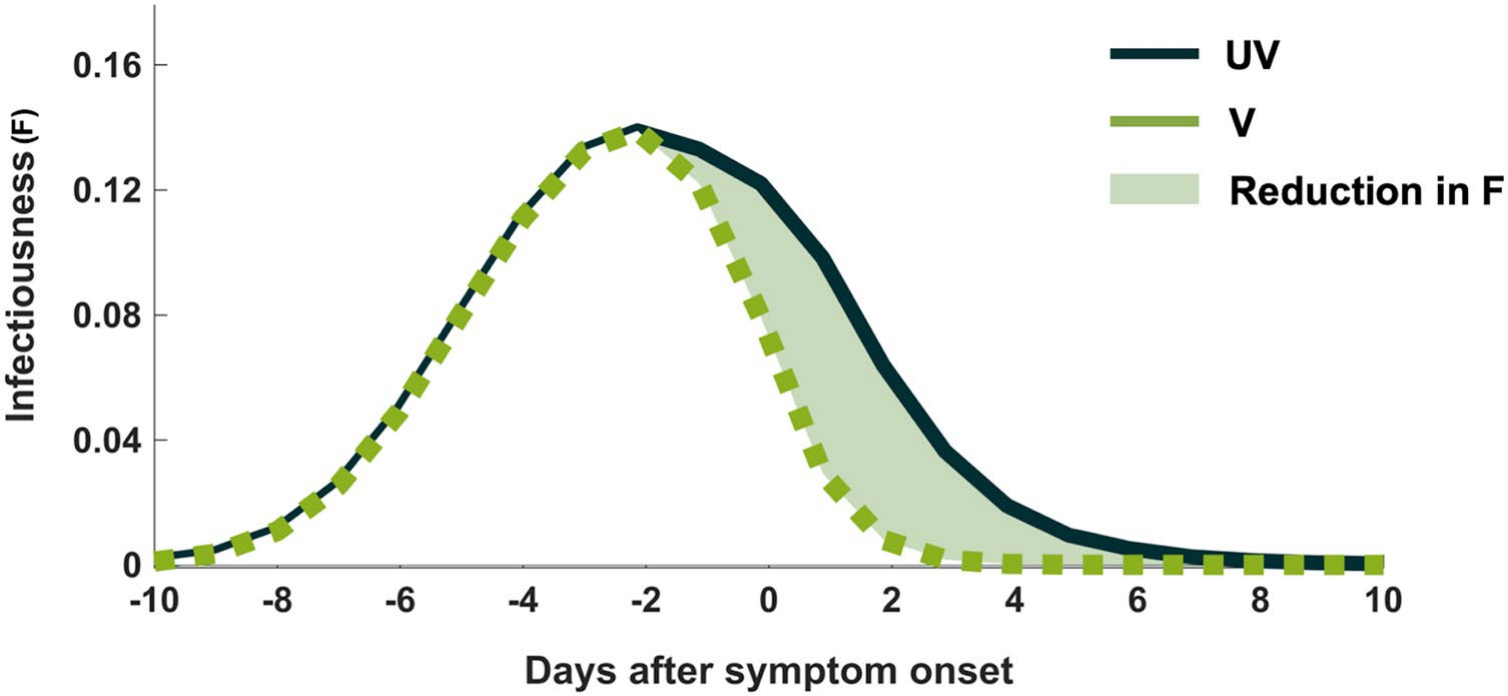
An illustration of the infectiousness profile for infections in unvaccinated individuals (UV) extracted from^18^ and the estimated infectiousness profile for vaccine breakthrough infections (V). The light green shade indicates a reduction in infectiousness (*F*) in vaccinated individuals due to faster viral clearance.

Infectiousness profiles describe the level of infectiousness of infected individuals during their infectious period. In this study, we used the infectiousness profiles of unvaccinated infected individuals estimated by Kang et al.^17,18^. The infectiousness of unvaccinated infected individuals peaks 2.1 days before the onset of symptoms and then decreases gradually during the course of the illness (Fig. 1).

For vaccine breakthrough infections, the infectiousness profile was estimated using two separate datasets. For the infectiousness profile during the virus clearance stage (after the peak viral load), the cycle threshold (*C*_*t*_) values of fully mRNA vaccine vaccinated infected individuals were extracted from a study in Singapore^19^. The *C*_*t*_ values were then converted to RNA copies or viral genome equivalents using the following equation^20^:

1 $${log}_{10}\left(\left[RNA\right]\right)=\frac{\left({C}_{t}-40.93733\right)}{\left(-3.60971\right)}+{log}_{10}\left(250\right)$$

where [RNA] denotes the RNA copies per milliliter (cp/ml), and -3.60971 and 40.93733 are the slope and intercept of the linear regression of *C*_*t*_ on the log_10_-transformed standard RNA concentration, respectively^20^. Infectiousness (*F*) was assumed to be directly proportional to the viral RNA genomes (*VL*) that exceeded a threshold of 10^6^ copies, i.e., *F ∝ VL × 10*^*–6*^^21^.

Recent studies revealed that there is no substantial difference in the mean peak height and proliferation duration of viral trajectories between vaccinated and unvaccinated individuals infected with the SARS-CoV-2 Delta variant^15,16^. Thus, in this study, the infectiousness profile during the proliferation stage (before the peak viral load) was assumed to have the same shape as the infectiousness profile of unvaccinated infected individuals obtained from Kang et al.^18^. Then, the infectiousness profile during the proliferation stage and the infectiousness profile during the virus clearance stage were combined to obtain the entire infectiousness profile of individuals infected after vaccine breakthrough (Fig. 1).

Although the viral trajectories during the proliferation stage are similar in both unvaccinated and vaccinated infected individuals, the viral loads are cleared faster in vaccine breakthrough infections than in unvaccinated individuals^15,16^. Because of the faster viral clearance time, the disease transmissibility of vaccinated infected individuals could be reduced compared to unvaccinated individuals. In this study, we evaluated vaccine effectiveness against transmission as a relative reduction in the infectiousness of vaccinated individuals (light green area in Fig. 1).

### Model structure

**Figure 2:**
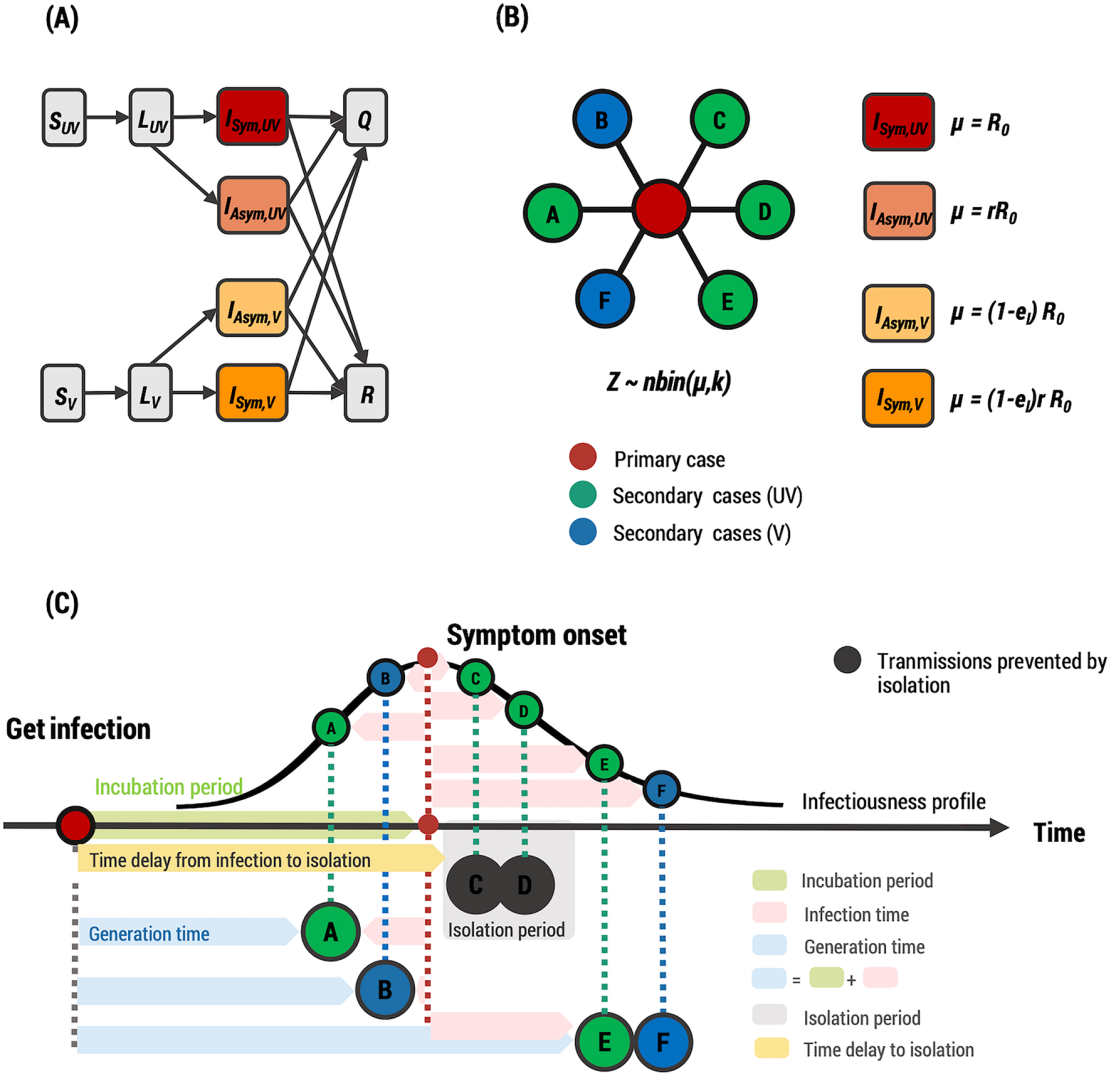
Model structure of COVID-19 transmission. (**A**) Schematic of the compartmental model showing the progression of the disease and transition of individuals across different compartments. (**B**) Example transmission network of a primary case (the red circle) and their expected secondary cases (the circles with letters A–F). The expected number of secondary cases was drawn from a negative binomial distribution with a mean $\mu$ and an overdispersion parameter *k *$(Z\sim nbin(\mu ,k))$*,* where $\mu$ takes a different value for infectious individuals in a different compartment, as shown in the right panel*.* (**C**) Example timeline of transmission events due to the primary case (the red circle) and their expected secondary cases (the circles with letters A–F). The probability of a secondary infection at time *t* was assumed to be proportional to the infectiousness of the infectious individuals at that time. The exposures of A and B occurred before the primary case was isolated. All expected transmission events during the isolation of the primary case were averted (gray circles); therefore, C and D were not infected. However, the primary case could still infect others after they were released from isolation. The incubation period, the time duration from exposure to symptom onset, was drawn from a gamma distribution. The generation time was the time duration between a primary case's infection and one of its subsequent secondary cases.

We developed an individual-based model of COVID-19 transmission to assess the impact of vaccination on the probability of post-isolation infections. The entire population was divided into those who had been fully vaccinated (*V*) and those who had not been vaccinated (*UV*). To simulate the transmission of the disease, individuals in both the *V* and *UV* groups were further categorized as susceptible (*S*), latent (*L*), infectious (*I*), isolated (*Q*), and recovered (*R*) according to their infection status. After being infected, individuals enter a latent state before becoming infectious. Finally, the infectious individuals moved either to the recovered or isolated groups. Infectious individuals were further divided into symptomatic (*I*_*Sym*_) and asymptomatic (*I*_*Asym*_) infectious individuals, with the assumption that asymptomatic infectious individuals are less infectious than symptomatic individuals^22^ (Fig. 2A). The subscripts *V* and *UV* are used to distinguish vaccine breakthrough infections from those in unvaccinated individuals.

Although COVID-19 vaccines cannot entirely protect people against infection, they are still beneficial in decreasing the chance of infection^14,23^. In addition, even when vaccinated individuals get infected, they will be less likely to transmit the disease to other individuals^24^ and also less likely to experience severe symptoms^25,26^. Individuals who have been fully vaccinated are less susceptible to the disease. In our model, we assumed that their infection probability was reduced by the vaccine effectiveness against infection (*e*_*S*_), i.e., the infection probability of vaccinated individuals is 1−*e*_*S*_ relative to those of unvaccinated individuals. In addition, when vaccinated individuals get infected, they have a lower chance of becoming symptomatic than unvaccinated people. So, their expected secondary infections, *Z*, were reduced by a fraction of 1−*e*_*I*_. In our model, we assumed that infections with SARS-CoV-2, regardless of their vaccination status, provide perfect immunity against re-infection during the time course of the simulation. The parameters and their default values used in the model are summarized in Table 1.

**Table 1:** Model parameters and their default values.

Parameter	Default value	Source
Basic reproduction number (*R*_*0*_)	5.08	^28^
Overdispersion parameter (*k*)	0.08	^29^
**Incubation period distribution (gamma distribution)**		
Mean	5.8 days	^17^
Shape parameter	3.64	
Scale parameter	1.59	
**Probability of being symptomatic**		
Unvaccinated individuals	0.573	^25^
Vaccinated individuals	0.431	^25^
Reduction in infectiousness of asymptomatic individuals (*r*)	0.58	^22^
Vaccine effectiveness against infection (*e*_*S*_)	0.79	^14^
Vaccine effectiveness against transmission (*e*_*I*_)	0.2455	Estimation
Probability that symptomatic individuals will be isolated	0.8	Assumption
Probability that asymptomatic individuals will be isolated	0.1	Assumption
**Time delay from infection to isolation**		
Primary case	0 days	Assumption
Other infected individuals in the community	6.8 days	Assumption

The number of secondary infections caused by a single primary case, *Z*, for each infected individual was estimated from a negative binomial distribution with a mean equal to the reproduction number (*R*_*0*_) and dispersion parameter (*k*) (Fig. 2B). Because of the lower infectivity of asymptomatic infectious individuals, they contribute to fewer infections; the mean number of secondary cases made by an asymptomatic infectious individual was reduced by a factor of *r*. In addition, the mean of the secondary infection distribution for the vaccine breakthrough infectious individuals was scaled by a factor of 1−*e*_*I*_^24–26^.

In our model simulation, we assumed that, initially, a certain fraction of 10^6^ individuals had been fully vaccinated and had vaccine-induced immunity. No one possessed immunity from prior infections. A primary case who was fully vaccinated was then imported. We assumed that the imported primary case had been isolated since their last recent exposure to the virus, while other subsequently infected individuals in the community were isolated with a default time delay of 6.8 days (estimated from the mean incubation period and the mean delay from symptom onset to isolation in South Korea^27^). An example of the transmission events is illustrated in Fig. 2(C). The incubation period, time from exposure to symptom onset, was assumed to follow the gamma distribution with a mean of 5.8 days^17^. After drawing *Z* from the secondary infection distribution, the time of each new secondary infection was drawn from a random number distribution that was distributed according to the infectiousness profile of the infectious individuals. Transmission events could only take place outside of the isolation period. Vaccinated individuals were less likely to become infected, with a reduction of 1-*e*_*S*_, because of vaccine effectiveness against infection. The effective infectious period was determined by whether infected individuals were isolated. If infected individuals were not isolated, they were contagious until they were isolated. The generation time between the infection of a primary case and one of its subsequent secondary cases was dependent on both the incubation period and the infection time.

### Estimating the probability of secondary transmission and a successful outbreak after isolation

Although secondary infections can be prevented during isolation of the primary case, post-isolation infections are still possible. The probability of post-isolation secondary transmission was defined as the chance that a primary case led to at least one subsequent infection after isolation. The probability of a successful outbreak was estimated from the likelihood that the chain of transmission initiated from the primary case after isolation continued for more than 90 days. We checked that the threshold value of 90 days can distinguish between simulations in which the disease goes extinct and simulations in which the disease spreads substantially until reaching the equilibrium state. The probabilities were estimated using three batches of simulations, each containing 1,000 model realizations.

## Results

### Impact of vaccination on post-isolation transmission

**Figure 3:**
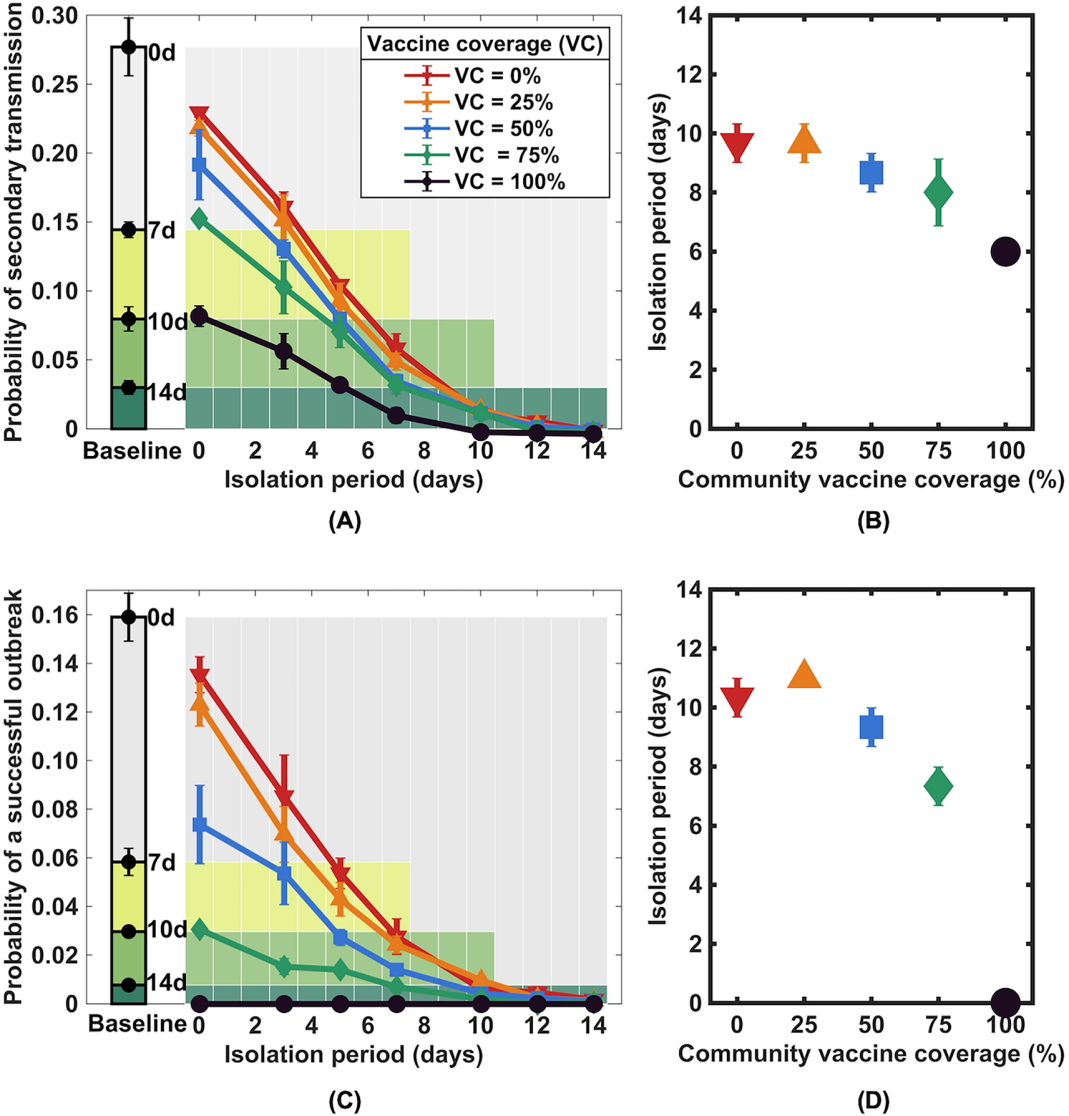
Impact of isolating a primary vaccinated infected individual on post-isolation transmission. Probability of secondary transmission (**A**) and probability of a successful outbreak in which a chain of transmission can be sustained (**C**) after a range of isolation periods and vaccination levels in the community. The corresponding probabilities in the baseline scenario where the primary case and all other individuals in the community are unvaccinated are shown as bar graphs on the left side of both subfigures. (**B**) and (**D**) show the isolation period equivalent to the 14-day isolation period in the baseline scenarios regarding the probability of secondary transmission and the probability of a successful outbreak, respectively. Error bars indicate 95% CIs.

We explored the probability of a primary infected individual leading to at least one secondary infection and the probability of a successful outbreak, i.e., having a sustained chain of transmission, after being released from isolation. In the baseline scenario in which the primary case and all other individuals in the community were unvaccinated, we found that there was an approximately 3% chance that the unvaccinated primary case would lead to at least one secondary infection after being isolated for 14 days, and a sustained chain of transmission could occur with a less than 1% chance (left bars in Fig. 3A,B). However, if the primary case had already been vaccinated, we found that although all other individuals in the community were unvaccinated, only approximately 10 days of isolation were equivalent to a 14-day isolation of an unvaccinated primary case (red lines and red symbols in Fig. 3).

Vaccinating people in the community can further reduce the likelihood of secondary infections and the probability of a successful outbreak. Higher community vaccine coverage decreased the chance of secondary transmission following the isolation of the vaccinated primary case, especially when the isolation periods were short. In addition, when the isolation period was longer than 12 days, there was no apparent difference between different vaccination coverage levels. At the outbreak risk equivalent to that of a 14-day isolation in the baseline scenario, the isolation duration of the primary vaccinated case could be shortened to 9.33 days (95% CI 8.68–9.98) if 50% of people in the community were vaccinated (Fig. 3D). When 75% of people in the community were vaccinated, the isolation period could be further shortened to 7.33 days (95% CI 6.68–7.98). Finally, we found that in the best-case scenario in which all individuals are vaccinated, although post-isolation infections are still possible for an isolation period of shorter than 6 days, the chance of a sustained chain of transmission occurring is extremely rare. In this case, isolation of infected individuals may no longer be necessary.

### Effect of waning vaccine-induced immunity

**Figure 4:**
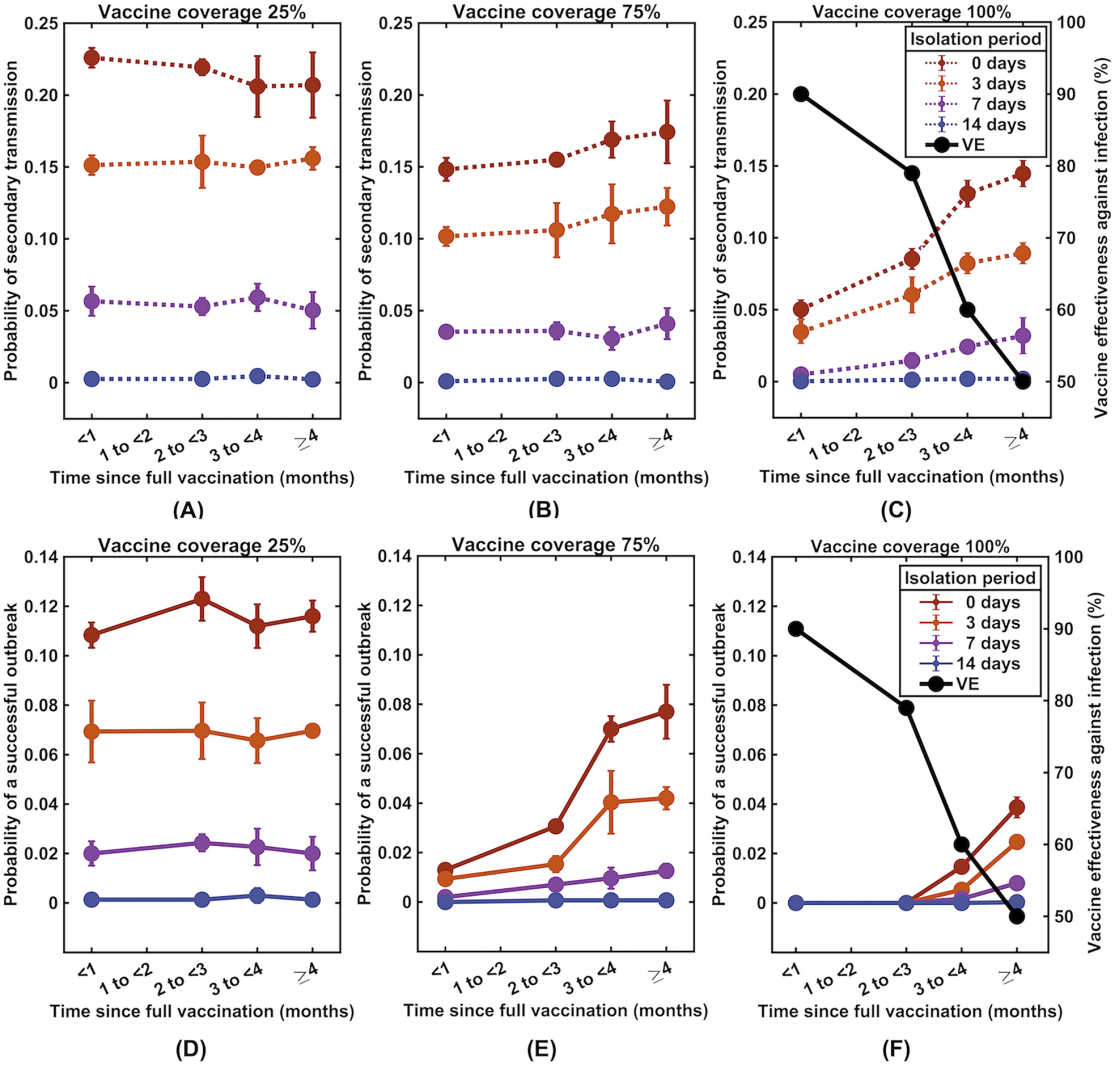
The effect of reductions in vaccine effectiveness against SARS-CoV-2 infection. The time evolution of the probability of at least one secondary infection (**A**–**C**) and probability of a successful outbreak **(D**–**F**) following the release of a breakthrough infectious individual from isolation as the vaccine effectiveness against infection wanes (black lines, right y-axis). Data on vaccine effectiveness against infection were obtained from reference^23^.

As vaccine effectiveness against Delta variant infection decreases over time^23^, we evaluated its effect on the probability of secondary infections and the probability of a successful outbreak following isolation. We found that for a low level of immunization (< 25% coverage), both the post-isolation transmission probability and the successful outbreak probability were not significantly affected by the waning of vaccine effectiveness (Fig. 4A and D). However, for higher vaccine coverage, the effect of the decline in vaccine effectiveness was more pronounced, especially when the isolation durations were short (Fig. 4B, C, E and F). Notably, with high vaccination coverage (> 75% coverage), there is a more substantial effect of immunity waning across a range of isolation periods, and the probability of an outbreak is still lower than that in the case when 25% of the population is vaccinated. With the vaccine coverage of 75%, for example, after four months of vaccination, the outbreak risk climbs from 0.9 to 4.2% for 3-day isolation and increases from 1.3 to 7.7% for no isolation (Fig. 4E). When all individuals in the community are vaccinated, despite a substantial decrease in vaccine effectiveness after four months, the chance of a successful outbreak is still lower than 4% even if there is no isolation (Fig. 4F).

**Figure 5:**
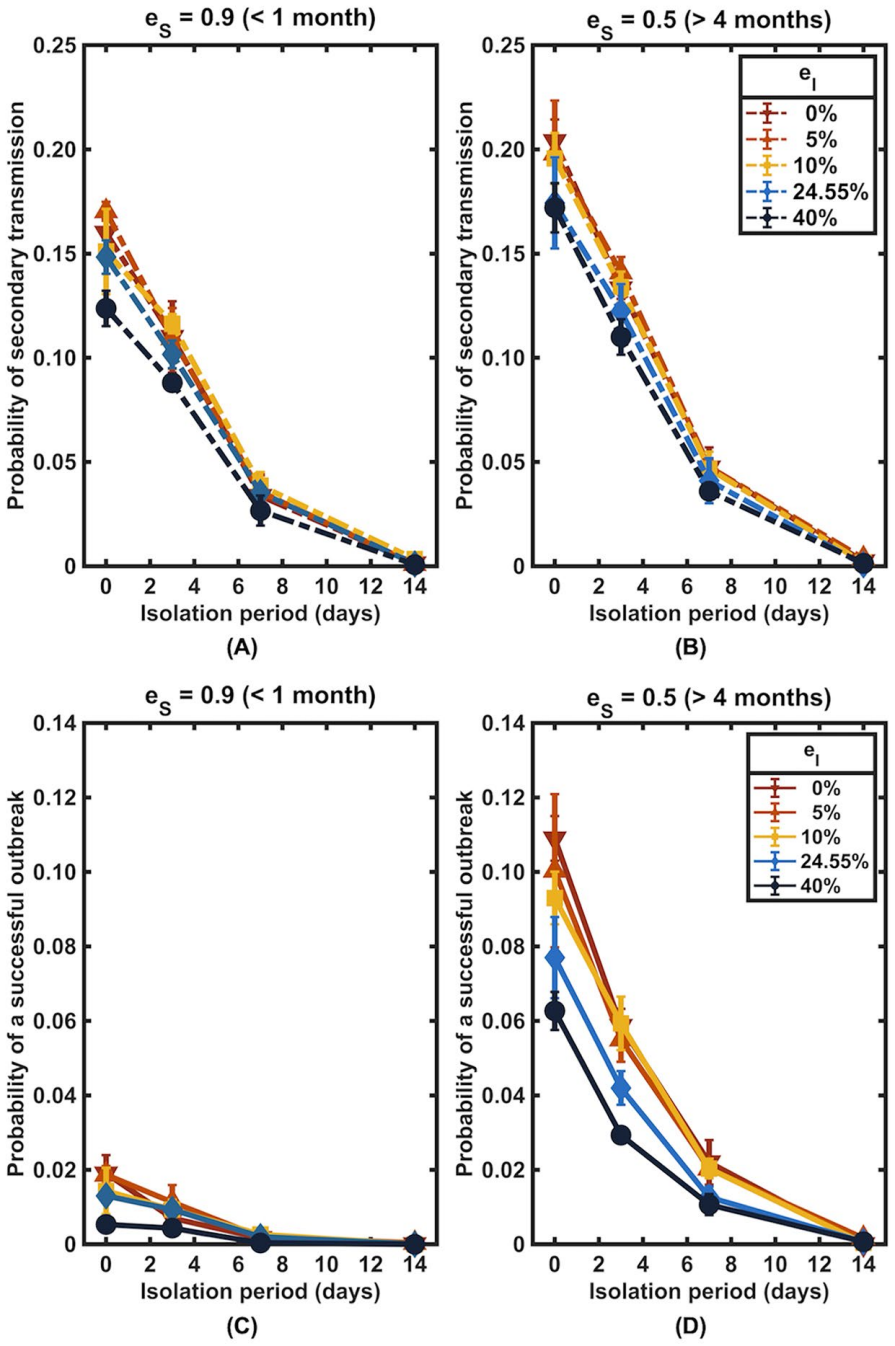
The influence of vaccine effectiveness against SARS-CoV-2 transmission. The probability of at least one secondary infection (**A** and **B)** and a successful outbreak (**C** and **D**) after being released from isolation into a community with a vaccination level of 75%. The vaccine effectiveness against transmission (e_I_) varied from 0 to 40%, and the vaccine effectiveness against infection (e_S_) was fixed at 90% (left column) and 50% (right column).

We also investigated how the change in vaccine effectiveness against transmission would influence the likelihood of secondary infections and the probability of a successful outbreak, considering the vaccine effectiveness against infection (*e*_*S*_) of 90% and 50%, corresponding to the effectiveness against infection of the Delta variant after being fully vaccinated with the BNT162b2 vaccine for one month and four months, respectively^23^. In this section, we considered that vaccine effectiveness against transmission (*e*_*I*_) ranged from 0 to 40%, reflecting different vaccine protection scenarios (waned and boosted) and different variants of SARS-CoV-2 (Delta and Omicron)^24^. We found that during the first four months after complete vaccination, when vaccine effectiveness against infection was as high as 90%, vaccine effectiveness against transmission had only a minor effect on the transmission, especially when the isolation period was long (Fig. 5).

### Impact of community case isolation and other control measures

**Figure 6:**
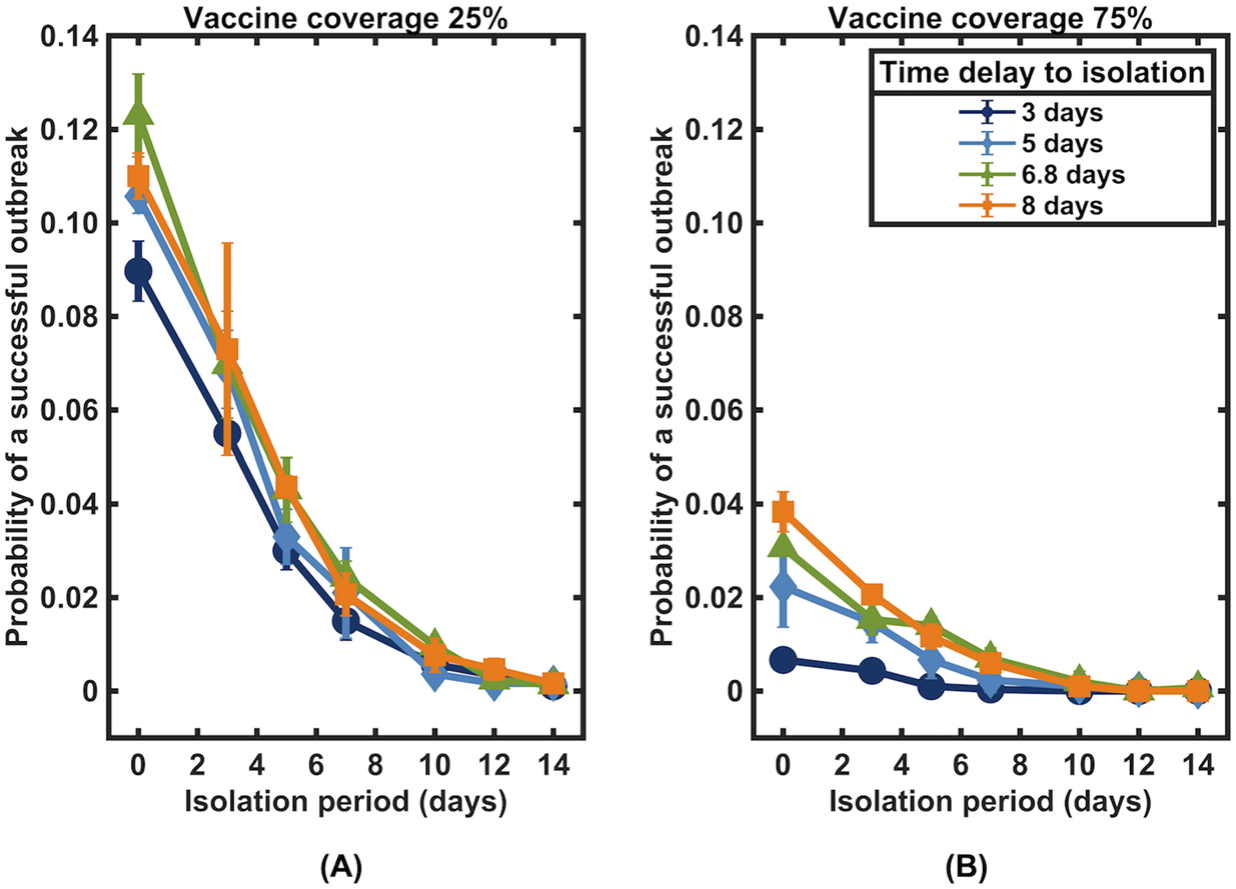
Impact of time delay from infection to isolation under vaccination coverage levels of (**A**) 25% and (**B**) 75%.

We next evaluated the impact of the time delay from infection to the isolation of infected individuals in the community on the spread of SARS*-*CoV*-*2. Our results indicated that the outbreak would be less likely to occur if case isolation was performed with a shorter delay (Fig. 6). For example, under vaccine coverage of 75%, the outbreak risk could be suppressed to lower than 1% if isolation could be performed within 3 days after infection. To maintain the same level of outbreak risk, a longer duration of isolation is needed for isolation with longer delays. For instance, for a 5-day delay, at least 5 days of isolation may be needed, and for a 7-day delay, at least 7 days of isolation may be needed. When only 25% of individuals are vaccinated, isolation may be required for at least 10 days, regardless of how quickly infected individuals are isolated.

**Figure 7:**
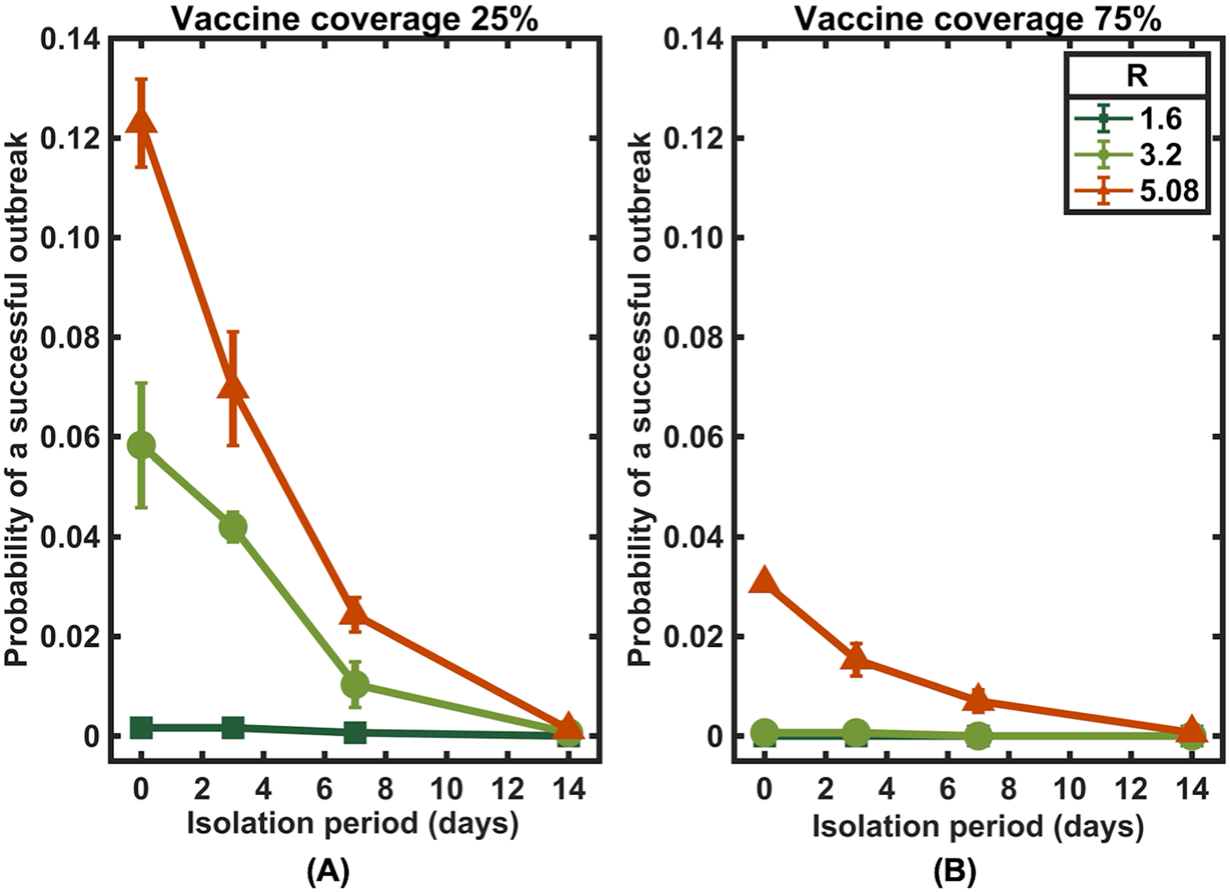
A sensitivity analysis of the effective reproduction number. The probability of a successful outbreak under community vaccination coverage levels of (**A**) 25% and (**B**) 75%.

The effective reproduction number (*R*) is commonly used to measure disease transmissivity under different control measures. To consider the effects of other control measures, a sensitivity analysis of the effective reproduction number was performed. In combination with other nonpharmaceutical interventions, we found that community vaccination could further shorten the isolation period (Fig. 7). For instance, in the absence of any nonpharmaceutical interventions and with vaccine coverage of only 25%, case isolation may be required for at least 12 days to reduce the outbreak risk to below 1%. However, if other control measures are concurrently implemented at a level that could reduce the effective reproduction number to 3.2, only one week of isolation is sufficient. Importantly, in this case, isolation will no longer be necessary if the community vaccination level reaches 75%.

## Discussion

In this work, we evaluated the likelihood of at least one secondary infection and the likelihood of an outbreak following the isolation of a vaccine breakthrough infectious individual for a specified period under different community vaccination coverage levels. Our modeling results indicated that vaccines play a critical role in reducing the likelihood of post-isolation transmission. In equivalent isolation durations to 14-day isolation in the scenario where no vaccine was available, we found that the duration of isolation for an infected individual who has already been fully vaccinated could be reduced to 10 days even though all other individuals in the community are unvaccinated. Additionally, the duration of isolation can be reduced further if the majority of the community members are immune to the disease (Fig. 3). In an ideal scenario in which all individuals in the community are fully vaccinated with two doses of mRNA BNT162b2 vaccine, isolation of Delta variant breakthrough infected individuals may no longer be needed, at least during the first three months after being fully vaccinated if no other nonpharmaceutical interventions are implemented. After three months, however, as vaccine effectiveness against infection drops to approximately 60%^23^, the probability of post-isolation transmission increases rapidly after this time, especially in the cases of short isolation periods. This result indicates that booster vaccination may be needed after being fully vaccinated for three months; otherwise, more extended isolation periods or other nonpharmaceutical control measures may be necessary to compensate for the increased transmission risk (Fig. 4). The post-isolation secondary infection probabilities across different scenarios are summarized in Table S1 in the Supplementary Material.

With a faster viral clearance time in vaccinated individuals, vaccines have been hypothesized to reduce onward transmission from vaccinated infected individuals. According to our estimations, the vaccine effectiveness against transmission of 24.6% is comparable to the effectiveness against transmission with the Delta variant after receiving two doses of the BNT162b2 vaccine^24^. However, the emergence of the Omicron variant has raised serious concerns about its capability to evade vaccine protection. After receiving two doses of mRNA vaccine, the vaccine effectiveness in preventing Omicron variant transmission drops to less than 5%^24^. Nevertheless, our simulations showed that reduced vaccine effectiveness against Omicron variant transmission does not greatly affect the risk of secondary infection if the vaccine effectiveness against infection is restored to a high level via booster vaccination^30^ (Fig. 5). However, since at the time of writing this manuscript, the data on the waning of vaccine effectiveness against Omicron variant infections were not available, we assumed that it wanes at the same rate as for the Delta variant.

When considering the effect of delays in isolating infected individuals in the community, we found that a shorter delay to isolation can further shorten the isolation period, especially in high vaccine coverage settings. In addition, we found that while an outbreak may still occur in the absence of isolation in the community with a low vaccination coverage level, the risk could be minimized when additional control measures such as contact tracing and quarantine of their contacts, as well as testing, are implemented to reduce the effective reproduction number (Figs. 6 and 7).

Regarding the likelihood of post-isolation secondary infection and the likelihood of an outbreak following the isolation of a vaccine breakthrough infected individual, our modeling results highlighted that the isolation period of infected individuals could be shortened once some individuals in the community have been vaccinated. These results would help policy-makers decide when to relax the isolation policy to limit related economic impacts. In addition, our results showed that the case isolation measure is more effective when performed promptly, suggesting that, in addition to vaccination, effective contact tracing and disease surveillance systems are essential in disease control.

Our study, however, has some limitations. First, we assumed that neither infection-acquired nor hybrid immunity existed in the initial population. Second, the model parameters used in this study were based on the Delta variant and the BNT162b2 vaccine, which might limit the applicability for the current COVID-19 situation in which the most prominent circulating variant of SARS-CoV-2 is the Omicron (BA.5) variant. Third, it was assumed that SARS-CoV-2 infections would provide perfect immunity against reinfection, which might be invalid if a new variant of SARS-CoV-2 emerges. Finally, isolation measures were assumed to be perfectly implemented, and everyone adhered to the regulations.

## Conclusions

We found that in the baseline scenario in which all individuals in the community are unvaccinated, there is a 3 percent chance that a primary case will lead to at least one secondary infection after being isolated for 14 days. In this case, a sustained chain of transmission can occur with a less than 1 percent chance. With an outbreak risk equivalent to that of 14-day isolation in the baseline scenario, we discovered that the isolation period could be shortened to 7.33 days (95% CI: 6.68–7.98) if, during the last three months, 75% of the community had been fully vaccinated with the BNT162b2 vaccine. In the best-case scenario, in which everyone in the community is fully vaccinated, isolating those infected with the Delta variant may no longer be necessary. However, a booster vaccination may be required three months after full vaccination to maintain the outbreak risk below 1%.

## Supplementary Information

Supplementary Table S1.

## Data Availability

The authors report that the data supporting the findings of this study are available within the paper.
