# Supplementary material for: Individual-based modeling reveals that the COVID-19 isolation period can be shortened by community vaccination
Source: Sci Rep. 2022 Oct 20;12:17543. doi: 10.1038/s41598-022-21645-y (PMC9583066; doi:10.1038/s41598-022-21645-y)
Supplement: Supplementary file 1 — Supplementary Table S1. [file 41598_2022_21645_MOESM1_ESM.pdf]

Supplementary Material:

Individual-based modeling reveals that the COVID-19 isolation period can be shortened by community vaccination

Chayanin Sararat[1,2], Jidchanok Wangkanai[1,2], Chaiwat Wilasang[1,2], Tanakorn Chantanasaro[1,2], and Charin Modchang[1,2,3,4*]

[1]Biophysics Group, Department of Physics, Faculty of Science, Mahidol University, Bangkok 10400, Thailand.

[2]Center for Disease Modeling, Faculty of Science, Mahidol University, Bangkok 10400, Thailand.

[3]Centre of Excellence in Mathematics, MHESI, Bangkok 10400, Thailand.

[4]Thailand Center of Excellence in Physics, Ministry of Higher Education, Science, Research and Innovation, 328 Si Ayutthaya Road, Bangkok 10400, Thailand.

* Corresponding author

E-mail: charin.mod@mahidol.edu

Table S1. The post-isolation secondary infection probabilities across different scenarios.

Parameters					Probability of secondary transmission (%)				Probability of a successful outbreak (%)				Figures
VC	R	TtoQ (days)	e_S (%)	e_I (%)	IP 0d	IP 3d	IP 7d	IP 14d	IP 0d	IP 3d	IP 7d	IP 14d	
**0%**	5.08	6.8	0.79	24.55	23.07	16.33	6.23	0.37	13.53	8.53	2.77	0.17	Fig. 3
**25%**	1.6	6.8	0.79	24.55	-	-	-	-	0.17	0.17	0.07	0.00	Fig. 7
	3.2	6.8	0.79	24.55	-	-	-	-	5.83	4.20	1.03	0.07	Fig. 7
	5.08	3	0.79	24.55	-	-	-	-	8.97	5.50	1.50	0.10	Fig. 6
		5	0.79	24.55	-	-	-	-	10.57	6.87	2.10	0.17	Fig. 6
		6.8	0.5	24.55	20.70	15.60	5.03	0.23	11.60	6.97	2.00	0.13	Fig. 4
			0.6	24.55	20.60	14.97	5.93	0.47	11.20	6.57	2.27	0.30	Fig. 4
			0.79	24.55	21.93	15.37	5.30	0.27	12.30	6.97	2.43	0.13	Figs. 3,4,6,7
			0.9	24.55	22.60	15.13	5.67	0.27	10.83	6.93	2.00	0.13	Fig. 4
		8	0.79	24.55	-	-	-	-	11.00	7.30	2.07	0.17	Fig. 6
**50%**	5.08	6.8	0.79	24.55	19.33	13.33	3.90	0.47	7.37	5.37	1.40	0.10	Fig. 3
**75%**	1.6	6.8	0.79	24.55	-	-	-	-	0.00	0.00	0.00	0.00	Fig. 7
	3.2	6.8	0.79	24.55	-	-	-	-	0.07	0.07	0.00	0.00	Fig. 7
	5.08	3	0.79	24.55	-	-	-	-	0.67	0.43	0.03	0.00	Fig. 6
		5	0.79	24.55	-	-	-	-	2.23	1.47	0.23	0.00	Fig. 6
		6.8	0.5	0	20.40	13.33	4.73	0.23	10.90	5.87	2.20	0.00	Fig. 5
				5	19.80	14.10	4.77	0.37	10.03	5.50	2.03	0.17	Fig. 5
				10	19.57	13.40	4.67	0.17	9.30	5.93	2.03	0.03	Fig. 5
				24.55	17.43	12.23	4.10	0.07	7.70	4.20	1.27	0.07	Figs. 4,5
				40	17.20	11.00	3.60	0.13	6.27	2.93	1.07	0.07	Fig. 5
			0.6	24.55	16.90	11.73	3.07	0.27	7.00	4.03	0.97	0.07	Fig. 4
			0.79	24.55	15.50	10.60	3.60	0.27	3.07	1.53	0.70	0.07	Figs. 3,4,6,7
			0.9	0	16.00	11.00	3.37	0.13	1.90	0.70	0.17	0.00	Fig. 5
				5	17.03	10.93	3.57	0.10	1.87	1.13	0.20	0.03	Fig. 5
				10	15.10	11.63	3.87	0.33	1.43	0.93	0.27	0.00	Fig. 5
				24.55	14.83	10.17	3.53	0.10	1.30	0.93	0.20	0.00	Fig. 4, 5
				40	12.37	8.80	2.67	0.07	0.53	0.43	0.03	0.00	Fig. 45
		8	0.79	24.55	-	-	-	-	3.83	2.07	0.60	0.00	Fig. 6
**100%**	5.08	6.8	0.5	24.55	14.47	8.93	3.20	0.20	3.87	2.47	0.80	0.03	Fig. 4
			0.6	24.55	13.07	8.23	2.43	0.20	1.47	0.53	0.17	0.00	Fig. 4
			0.79	24.55	8.53	6.03	1.47	0.13	0.00	0.00	0.00	0.00	Figs. 3,4
			0.9	24.55	5.03	3.47	0.50	0.03	0.00	0.00	0.00	0.00	Fig. 4

VC = Vaccine coverage,

TtoQ = Time delay from infection to the isolation,

IP = Isolation period.
